# Chronic Intestinal Pseudo-Obstruction and Lymphoproliferative Syndrome as a Novel Phenotype Associated With Tetratricopeptide Repeat Domain 7A Deficiency

**DOI:** 10.3389/fimmu.2019.02592

**Published:** 2019-11-07

**Authors:** Marie-Thérèse El-Daher, Julie Lemale, Julie Bruneau, Claire Leveau, Frédéric Guerin, Nathalie Lambert, Jean-Sébastien Diana, Bénédicte Neven, Fernando E. Sepulveda, Aurore Coulomb-L'Hermine, Thierry Molina, Capucine Picard, Alain Fischer, Geneviève de Saint Basile

**Affiliations:** ^1^Laboratory of Normal and Pathological Homeostasis of the Immune System, INSERM UMR 1163, Paris, France; ^2^Imagine Institute, Université de Paris, Paris, France; ^3^Pediatric Nutrition and Gastroenterology Department, Trousseau Hospital, Assistance Publique-Hôpitaux de Paris, Sorbonne Université, Paris, France; ^4^Department of Pathology, Hôpital Necker-Enfants Malades, Assistance Publique des Hôpitaux de Paris, Paris, France; ^5^Center for the Study of Primary Immunodeficiencies, Necker Enfants Malades Hospital, Assistance Publique des Hôpitaux de Paris, Paris, France; ^6^Pediatric Hematology Department, Hôpital Necker-Enfants Malades, Assistance Publique-Hôpitaux de Paris, Paris, France; INSERM UMR 1163, Paris, France; ^7^Centre Nationale de la Recherche Scientifique – CNRS, Villejuif, France; ^8^Department of Pathology, Hôpital A Trousseau, Assistance-Publique des Hôpitaux de Paris, Sorbonne Université, Paris, France; ^9^Laboratory of Lymphocyte Activation and Susceptibility to EBV Infection, INSERM UMR 1163, Paris, France; ^10^Collège de France, Paris, France

**Keywords:** tetratricopeptide repeat domain 7A, lymphoproliferative syndrome, intestinal pseudo-obstruction, *fsn* mouse, mutant's cell phenotype

## Abstract

Mutations in the tetratricopeptide repeat domain 7A (TTC7A) gene cause very early onset inflammatory bowel diseases (VOIBD) or multiple intestinal atresia associated with immune deficiency of various severities, ranging from combined immune deficiency to mild lymphopenia. In this manuscript, we report the clinical, biological and molecular features of a patient born from consanguineous parents, presenting with recurrent lymphoproliferative syndrome and pan-hypergammaglobulinemia associated with chronic intestinal pseudo obstruction (CIPO). Genetic screening revealed the novel c.974G>A (p.R325Q) mutation in homozygosity in the *TTC7A* gene. The patient's phenotype differs significantly from that previously associated with TTC7A deficiency in humans. It becomes closer to the one reported in the ttc7a-deficient mice that invariably develop a proliferative lymphoid and myeloid disorder. Functional studies showed that the extreme variability in the clinical phenotype couldn't be explained by the cellular phenotype. Indeed, the patient's TTC7A mutation, as well as the murine-ttc7 mutant, have the same functional impact on protein expression, DNA instability and chromatin compaction, as the other mutations that lead to classical TTC7A-associated phenotypes. Co-inheritance of genetic variants may also contribute to the unique nature of the patient's phenotype. The present case report shows that the clinical spectrum of TTC7A deficiency is much broader than previously suspected. Our findings should alert the physicians to consider screening of *TTC7A* mutations in patients with lymphoproliferative syndrome and hypergammaglobulinemia and/or chronic intestinal pseudo-obstruction.

## Introduction

In humans, biallelic loss-of-function mutations in tetratricopeptide repeat domain 7A (TTC7A) have been shown to cause intestinal and immune disorders of variable severity. The disease phenotype ranges from very-early-onset inflammatory bowel disease (VOIBD) to multiple intestinal atresia, accompanied by mild lymphopenia (ELA) or even combined immunodeficiency (MIA-CID, respectively) (1–4). The TTC7A protein is a member of the tetratricopeptide repeat family and is thought to interact with the components of many different macromolecular complexes. For example, TTC7A reportedly interacts with the phosphatidylinositol 4-kinase alpha (PI4K), which catalyzes the production of phosphatidylinositol 4-phosphate at the plasma membrane, and the protein EFR3 homolog B (EFR3), which may serve as a membrane anchor for PI4K (3, 5). Furthermore, TTC7A downregulates the RhoA-ROCK signaling pathway and the latter's downstream targets involved in actin dynamics (2, 4). Lastly, it was recently reported that a high proportion of the cell's TTC7A is located in the nucleus, where the protein modulates transcription and chromatin structure (6). Hence, TTC7A have many functions, but the pathophysiological mechanisms underlying the disorders presented by TTC7A-deficient patients have yet to be fully characterized.

## Case Report

**Figure 1:**
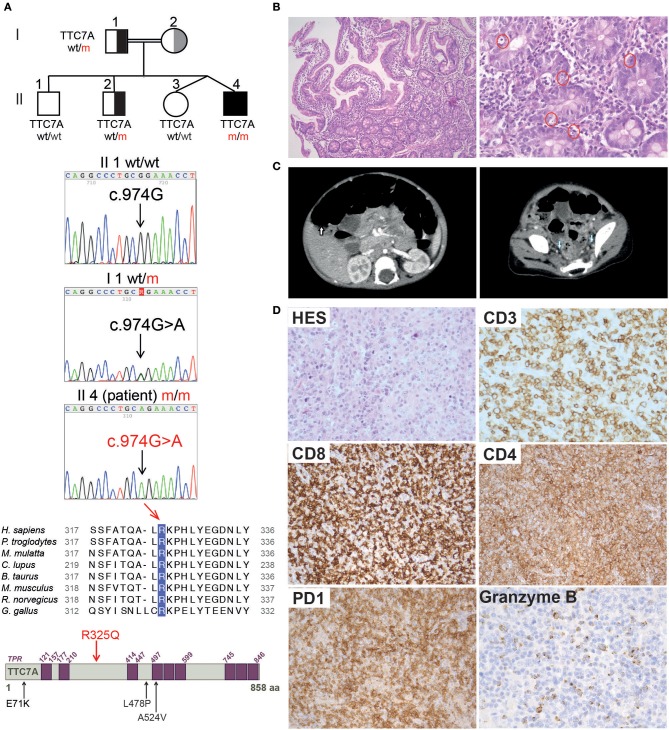
Family pedigree, TTC7A mutation, and histopathological findings in reported patient. **(A)** From top to bottom: family pedigree; Sanger sequencing chromatogram depicting the c.974G>A mutation in *TTC7A* gene; alignment of the amino acid (aa) surrounding the conserved R325 residue in TTC7A homologs from 8 species; illustration of TTC7A protein in which purple boxes indicate tetratricopeptide repeats (TPRs) domains, the R325Q homozygous mutation identified in reported patient (Il4 m/m) is highlighted in red, in contrast to mutations previously reported in other TTC7A patients and relevant to this study, which are indicated in black. **(B)** Histologic analysis of duodenal biopsies from P_R325Q patient (11 months old). Hematoxylin and Eosin (H&E) staining was performed, magnifications of the left and right panels are 100 and 400X, respectively. In the higher magnification panel, red circles denote excess apoptotic epithelial cells in the crypts. **(C)** Scanners showing hepatosplenomegaly (left, white arrow) and lymphadenopathies (right, blue arrow) performed in P_R325Q patient at the age of 14 months. **(D)** Histologic analysis of axillary lymph nodes from P_R325Q patient at 6.5 years. H&E staining (HES) (top left, 400X magnification) of a T cell area showed a polymorphic expansion of lymphoid cells admixed with plasma cells, macrophages and eosinophils. CD3 (400X magnification) positive T cells were often medium and large. These T cells were predominantly CD8, with expression of PD1 (200X magnification) and granzyme B (400X magnification). CD4 staining (200X magnification) identified numerous macrophages and a minority of lymphocytes.

Here, we report on a male patient born at 37 weeks of gestation in a twin pregnancy (birth weight: 2730 g; birth length: 47 cm) to consanguineous parents of Algerian descent (Figure 1A). Soon after birth, the infant displayed abdominal bloating, frequent liquid stools, and failure to thrive. Neither vomiting nor rectal bleeding were observed. The results of clinical examination were otherwise unremarkable. The replacement of breast milk by an extensive protein hydrolysate did not lead to an improvement. At the age of 11 months, the infant was hospitalized following an aggravation of the digestive symptoms that necessitated parenteral nutrition. The results of a sweat test, abdominal ultrasound examination, serologic assay (for HSV, CMV, EBV, and *mycoplasma*), a urine catecholamine test and auto-immune investigations were all negative. Gastroduodenal and rectal endoscopy did not reveal any abnormal macroscopic features. A histology assessment highlighted the presence of non-specific proctitis and gastro-duodenitis, characterized by a moderate inflammatory infiltrate containing a high proportion of mononuclear cells and a few eosinophils (Figure 1B). The duodenal crypt epithelium showed excessive apoptosis. Sections of antrum were negative for *Helicobacter pylori*. A diagnosis of chronic intestinal pseudo-obstruction (CIPO) was suggested by the impaired upper digestive tract function, segmental dilatation of the jejunum and ileum, an atypical rectoanal inhibitory reflex. The diagnosis was confirmed on a rectal surgical biopsy that showed numerous submucosal nerves and myenteric plexus of variable size with many ganglion cells (Supplementary Figure 1). Hair microscopical analysis revealed no anomalies including absence of trichorrhexis nodosa.

At the age of 14 months, the patient presented with prolonged fever complicated by acute respiratory distress syndrome (ARDS), multiple lymphadenopathies (in the axillary, inguinal, mediastinal, and retroperitoneal areas) and hepatosplenomegaly (Figure 1C). A histologic assessment of an axillary lymph node showed a slight hyperplasia of the T cell zone, mostly consisting in small CD4^+^ T lymphocytes, together with histiocytes and dendritic cells, although the overall architecture was unaffected (data not shown). Immunologic profiling of the patient's peripheral blood mononuclear cells revealed moderate CD8+ and NK lymphopenia, a normal CD4^+^ lymphocyte count, normal lymphocyte proliferation in responses to mitogens and tetanus toxoid antigens, and hypergammaglobulinemia (IgG, IgA, IgM, and IgE) (Table 1 and data not shown). The patient improved after the initiation of corticosteroid therapy and then azathioprine that was given for 12 months. The patient experienced a CMV infection and severe varicella 6 months later, both with a favorable outcome. Despite the persistence of intestinal bloating, the recovery of normal oral feeding with relatively normal stools made it possible to discontinue parenteral nutrition at the age of 2.

**Table 1:** Immunological phenotypes of TTC7A patient carrying R325Q homozygous mutation.

**Lymphocytes**	**Age 17 months**	**Age 6.5 years**
**CELL COUNTS, CELLS/μl (NORMAL RANGE)**		
CD3^+^ T cells	2,940 (2,100–6,200)	2,950 (1,200–2,600)
CD4+	2,156 (1,300–3,400)	1,578 (650–1,500)
CD8+	588 (620–2,000)	1,327 (370–1,100)
CD45RA^+^CD4^+^%	55 (73–86)	49 (58–70)
CCR7^+^CD45RA^+^/CD8^+^%	N/A	7 (52–68)
CCR7^+^CD45RA^−^/CD8^+^%	N/A	2 (3–4)
CCR7^−^CD45RA^−^/CD8^+^%	N/A	68 (11–20)
CCR7^−^CD45RA^+^/CD8^+^%	N/A	23 (16–28)
CD19^+^ B cells	1,764 (720–2,600)	251 (273–860)
CD27^+^/CD19^+^%	N/A	11 (8.1–33.3)
CD27^−^IgD^+^/CD19^+^%	N/A	88 (59.7–88.4)
CD27^+^IgD^+^/CD19^+^%	N/A	1 (2.9–17.4)
CD16^+^CD56^+^ NK cells	98 (180–920)	143 (100–480)
	**Age 17 months**	**Age 6.5 years**
**CELL COUNTS, CELLS/ μl (NORMAL RANGE)**		
**Polymorphonuclear**		
- Neutrophils	10,500 (1,500–8,500)	12,900 (1,800–8,000)
- Eosinophil	3,000 (0–300)	800 (0–300)
Monocytes	1,000 (200–1,000)	1,800 (200–1,000)
Platelets	503,000(175,000–500,000)	956,000(175,000–420,000)
Hemoglobin g/dl	11.7 (10.5–12)	9.4 (11.5–13.5)
**Immunoglobin**	**Age 17 months**	**Age 6.5 years**
**SERUM IMMUNOGLOBIN LEVELS (NORMAL RANGE)**		
IgG g/L	13.24 (4.57–8.49)	26.83 (5.82–11.54)
IgA g/L	0.87 (0.27–0.86)	9.89 (0.46–1.57)
IgM g/L	7.06 (0.47–1.31)	9.06 (0.54–1.55)
IgE UI/ml	12 000 (<114)	N/A

*N/A, not available*.

At the age of 6 and a half, 3 months after the withdrawal of corticosteroid therapy, the patient's lymphoproliferative syndrome relapsed. A histologic assessment of several lymphadenopathies showed an important expansion of the T cell area with a polymorphic infiltrate made of medium to large-sized lymphoid cells, plasma cells, eosinophils and macrophages, associated with centrofollicular B lymphoid depletion. T lymphocytes were mainly CD3^+^CD8^+^ differentiated lymphocytes expressing granzyme B and PD1 (Figure 1D). There was no evidence of Epstein-Barr Virus (EBV) infection by Epstein-Barr virus–encoded small RNA (EBER) *in situ* hybridization. A bone marrow biopsy highlighted a moderate plasma cell hyperplasia and an excess of interstitial CD8^+^ T lymphocytes. Immunologic profiling evidenced marked hypergammaglobulinemia of all isotypes tested (IgG, IgA, IgM) that was associated with moderate CD4^+^ and CD8^+^ T lymphocytosis, a low proportion of naïve CD4^+^ and CD8^+^ T lymphocytes, a high proportion of CD8^+^ memory T lymphocytes, and moderate B cell lymphopenia (Table 1). Reintroduction of azathioprine led to full resolution of the lymphoproliferative syndrome. Two years later, the patient is still on azathioprine maintenance therapy, and there are no clinical signs of lymphoproliferation.

## Genetic and Functional Characterization of TTC7A-Deficiency

Analysis with 301-gene primary immunodeficiency (PID) panel and then Sanger sequencing revealed a novel homozygous mutation in *TTC7A* exon 7 of (NM_020458.3: c.974G>A) on chromosome 2, leading to p.R325Q (Figure 1A). The patient's father and one of the three healthy siblings were heterozygous for the mutation and the two remaining siblings were wild type (Figure 1A and Supplementary Figure 2). Samples from the mother were not available. A segregation analysis of polymorphic markers spanning the TTC7A locus identified a homozygous region in the patient and was in accordance with the mutation segregation in the family (Supplementary Figure 2). The mutation affects a highly conserved amino acid at position 325 in the TTC7A protein (Figure 1A). The overall minor allele frequency (MAFs) is 0.00024 in gnomAD and the mutation is predicted to be pathogenic by *in silico* soft-wares (PolyPhen-2 (score: 0.07), SIFT (0.01) and CADD (24.3)). Based on the literature data and our findings, we considered that a novel homozygous mutation in *TTC7A* was the most plausible cause of patient's disease.

**Figure 2:**
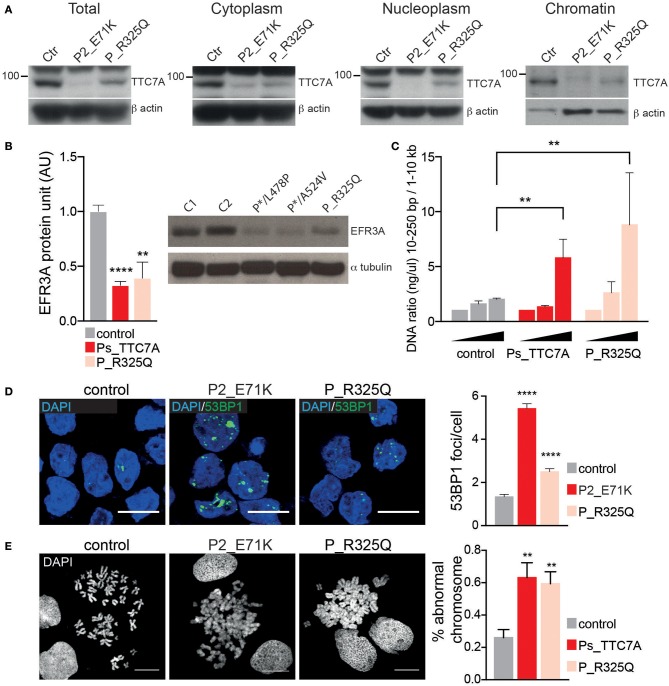
Phenotypic consequences of R325Q mutation. **(A)** The expression level of TTC7A protein (96 kDa) is decreased in the different cellular compartments extracted from B lymphoblastoid cell lines (B-LCLs) from R325Q_TTC7A (P_R325Q) patient and from TTC7A-deficient patient (P2_E71K) as compared to healthy donors (Ctr). β actin was used as an internal loading control in the western blot. **(B)** The expression level of the protein EFR3 homolog A (EFR3A) is decreased in B-LCLs from R325Q_TTC7A patient and from TTC7A-deficient patients (P*/L478P and P*/A524V), as compared to control. The level of EFR3A protein was determined by western bot and the graph represents average level from three independent experiments, error bars indicate standard error of the mean (± SEM). Previously reported TTC7A patients are indicated as Ps_TTC7A when grouped. Unpaired *t*-test; **p = 0.002, ****p < 0.0001. **(C)** DNA accessibility is increased in B-LCLs from P_R325Q and from Ps-deficient patients (P1_E71K, P2_E71K, P*/L478P) as compared to control. The concentration of digested DNA was measured at different time points and the ratio between small and large size DNA fragments are shown in the graph. The graph indicates the average of DNA ratio measured from four independent experiments (± SEM), Mann-Whitney test, **p < 0.01. **(D)** DNA damage is increased in patients' B-LCLs as compared to control, with an accumulation of 53BP1 foci. Left: immunostainings of 53BP1 imaged by confocal microscopy, Right: quantification of 53BP1 foci number. Graph represents the average count obtained from two independent experiments (Mean ± SEM). Total number of cells in control = 508; P2_E71K *n* = 315; P_R325Q *n* = 452; Sidak's multiple comparisons test, ****p < 0.0001. **(E)** Impaired chromatin condensation of patients' B-LCLs as compared to control. Left: chromosomes are stained with DAPI and imaged by confocal microscopy. Right: quantification of chromosomes with abnormal structure was performed on four independent experiments and the average count is shown in the graph (± SEM). Unpaired *t*-test, *p* = 0.0076 for Ps_TTC7A (P1_E71K, P2_E71K and P*/L478P) vs. control, and *p* = 0.006 for P_R325Q vs. control. Scale bar: 10 microns.

The phenotype of the patient described here differs significantly from that previously associated with TTC7A deficiency in humans; indeed, some of the features contrasted sharply. In particular, the patient presented with recurrent lymphoproliferative syndrome and pan-hypergammaglobulinemia but there was no evidence of disruption of the digestive tract's mucosal architecture, while CIPO was diagnosed. Hence, we decided to assess the functional consequences of the identified TTC7A mutant (p.R325Q). As seen with most mutants studied to date, TTC7A protein expression level of the R325Q mutant was lower in B lymphoblastoid cell line (Figure 2A) and in T lymphoblasts (Supplementary Figure 3A); the nucleoplasmic and chromatin-bound fraction were particularly low. Thus, the TTC7A-R325Q mutant protein appeared to be unstable leading to partial protein degradation, as seen for other reported mutants. The TTC7A protein stabilizes subunits like EFR3 in the complex that modulates the phospholipid composition at the plasma membrane (5). Accordingly, we found that patient's B cells expressed very low levels of EFR3 (Figure 2B). We recently showed that TTC7A in the nucleus regulates DNA accessibility, DNA stability, and chromatin condensation (6). By using micrococcal nuclease digestion assay and accumulation of 53BP1 foci, we observed a time-dependent increased in sensitivity to digestion and the spontaneous formation of many discrete 53BP1 foci in R325Q mutant cells, just as is seen in TTC7A-deficient cells from patients carrying other mutations (Figures 2C,D, Supplementary Figures 3B,C) (6). The TTC7A-R325Q mutant also had a detectable impact on chromatin condensation; the chromatin in B cell metaphases had a swollen, fuzzy appearance, as observed for previously reported TTC7A mutants with ELA and MIA-CID phenotypes (Figure 2E) (6). Thus, patient's unexpected clinical and immunological phenotype corresponds to a recurrent lymphoproliferative syndrome associated with a loss-of-function mutation in TTC7A. On the subcellular level, the functional characteristics of the R325Q mutant are the same as those seen for TTC7A mutants associated with the ELA and MIA-CID phenotypes. We looked for additional genetic alterations that might have modified patient's clinical phenotype. Using whole-exome sequencing, we identified an additional homozygous mutation in Ski2 like RNA helicase (*SKIV2L*) gene on chromosome 6 (c.1447C>T leading to p.Arg483Cys). This mutation was absent or heterozygous in the healthy family members. SKIV2L deficiency results in tricohepatoenteric syndrome (THES) (7). The homozygous c.1447C>T *SKIV2L* mutation was previously reported in a patient of African descent, displaying some of the features of THES (i.e., intrauterine growth retardation, VOIBD, liver disorders and immune defects) but lacking others (i.e., facial dysmorphism, skin and hair abnormalities, and cardiac defects) (7). With the exception of VOIBD, our patient did not display features of THES. Thus, the additional SKIV2L alteration is unlikely to account for our patient's clinical phenotype. However, we cannot rule out the possibility whereby, co-inheritance of this second genetic change contributes to the unique nature of the patient's phenotype relative to other reported cases of TTC7A-deficiency. Co-inheritance of genetic modifiers is increasingly been detected, thanks to next-generation sequencing, and can broaden phenotype heterogeneity (8). This has been recently illustrated in a patient carrying two homozygous mutations in the myeloid differentiation primary response protein 88 (MYD88) and caspase-associated recruitment domain-containing protein 9 (CARD9) genes (9), or in the interferon alpha receptor 1 and 2 (IFNAR1 and IFNGR2) genes (10), who presented with atypical phenotype considering the phenotype associated with each of the altered genes.

Interestingly, patient's immune phenotype is reminiscent of that observed in the Ttc7a-deficient *fsn* mouse. *Fsn* mice invariably develop a proliferative lymphoid and myeloid disorder, with hyperplasia of the spleen and lymph nodes (11). We thus assessed the functional consequences of Ttc7a deficiency in *fsn* splenocytes. We similarly found that *Ttc7a* mutation impaired Ttc7a protein expression, increased DNA accessibility and instability and modified chromosome compaction (Supplementary Figure 4). Reduction of EFR3 expression was previously reported in this murine model, further highlighting the cross-species conservation of the TTC7A's interactions and function (5). The mechanism(s) underlying phenotypic variability in these different contexts of TTC7A deficiency remains difficult to understand. Given TTC7A's broad subcellular distribution (i.e., at the plasma membrane, in the cytoplasm and nucleus) and many molecular interactions (2, 3, 5, 6) that converge to regulate cell homeostasis, we suggest each mutant can differentially alter specific protein-protein interactions, and thus lead to marked differences between the patients' phenotypes.

## Concluding Remarks

To date, TTC7A deficiency has been associated with intestinal disorders of variable severity (early and severe bloody diarrhea, apoptotic enterocolitis and atresia or inflammatory bowel disease), and immune phenotypes that range from severe lymphopenia and pan-hypogammaglobulinemia to the absence of immunodeficiency. The present case report shows that the clinical spectrum of TTC7A deficiency is even broader than previously suspected. Our findings should alert the physicians to consider screening of *TTC7A* mutations in patients with chronic intestinal pseudo-obstruction and/or lymphoproliferative syndrome and hypergammaglobulinemia. As far as we can tell, variability in the clinical phenotype is not explained by the cellular phenotype.

## Materials and Methods

### Informed Consent

Genetic studies and data collection procedures were approved by the local investigational review board and the French Advisory Committee on Medical Research (IRB registration # 00001072, n°ID-RCB/EUDRACT:2015-A01170-49). The family gave written informed consent for the publication of this case report. The results provided were kept anonymous. Patients with the E71K, P^*^/L478P, and P^*^/A524V mutations have been previously reported (4, 6), all of whom had given their consent to participation in the study.

### Patient Cell Culture

Blood samples were collected from the patients and control subjects. Peripheral Blood mononuclear cells (PBMCs) were isolated by density gradient centrifugation using Ficoll-Paque. B-lymphoblastoid cell lines (B-LCLs) transformed with EBV as described previously (2). Primary T lymphocytes were stimulated for 2 days with phytohemagglutinin (PHA) (5 μg/ml; Sigma-Aldrich, St Louis, Mo) and human Interleukin-2 (IL-2) (50–200 U/mL; PeproTech, Rocky Hill, NJ) in Panserin medium (PAN-Biotech GmbH, Aidenbach, Germany) supplemented with 10% human AB serum (S4190-100 eurobio life science). Cells were expanded up to 7–12 days after activation in full media containing 100U IL2. B-LCLs were cultured in RPMI medium 1640+Glutamax (Gibco, ref 61870-010) supplemented with 10% Fetal Bovine Serum (Gibco), sodium pyruvate (1 mM, LifeTechnologies) and penicillin and streptomycin (100 U/ml, LifeTechnologies).

### Immunohistochemistry

For morphological studies, duodenum and lymph node paraffin-embedded sections were stained with H&E. Immunostaining of lymph node section was performed as previously reported (2) with primary antibodies rabbit anti-CD3 (polyclonal, Dako), mouse anti-CD4 (clone 4B12, Thermo Scientific), mouse anti-CD8 (clone C8/144B, Dako), mouse anti-PD1 (NAT105, Abcam) and mouse anti-granzyme B (clone NCL, Leica).

### Ttc7a-deficient Mice and Cell Culture

Bone marrow-derived dendritic cells (BMDC) from homozygous Ttc7a *fsn*/*fsn* mice and their respective control littermates were obtained as previously reported. T splenocytes were stimulated with coated 2 μg/ul anti-CD3 (Clone 500A2, Ref 553238, BD) and soluble 1 μg/ul anti-CD28 (100 IU/mL; PeproTech, Rocky Hill, NJ) for 2 days. Cells were cultured in DMEM GlutaMax (Invitrogen, Ref 31966-021), Fetal Bovine Serum (Gibco), sodium pyruvate and MEM NEAA (1 mM, LifeTechnologies), penicillin/streptomycin (100 U/ml, LifeTechnologies), B-mercaptoethanol (1/1000, Gibco, Ref 31350-010), and human Interleukin-2 (100 U/ml). Cells were expanded up to 8–12 days after activation.

### Antibodies

Antibodies for immunoblots were obtained from the following commercial sources: αtubulin (abcam ab80779), 53BP1 (novus NB100-304), beta actin (genetex, GTX629630) and EFR3A (Sigma Ab2). TTC7A primary antibody has been previously described (4). Secondary antibodies used are Horseradish peroxidase coupled goat anti-Rabbit or mouse IgG (H + L) or are coupled to Alexa Fluor 488 and purchased from molecular probes (ThermoFisher).

### Protein Fractionation, Immunoprecipitation, and Immunoblotting

Subcellular fractionation enabled stepwise separation of cytoplasmic, membrane, nuclear soluble and chromatin-bound proteins were performed as previously reported (6) by using the ThermoFisher Kit (78840), following manufacturer's instructions.

Immunoprecipitations and immunobloting of Ttc7a in BMDCs was done as previously described (6) and according to standard procedure.

### Immunocytochemistry

Cells were deposited on poly-L-lysine coated coverslip and fixed with 4% paraformaldehyde in PBS for 20 min. Fixed cells were treated with 0.1 M glycine for 30 min, permeabilized with 0.5% Triton X-100 in PBS for 15 min. 53BP1 antibody (1/200) was incubated at room temperature for 1 h. Coverslips were washed and secondary antibody was incubated for 1 h. Cells were counterstained with DAPI and mounted with proLong Gold anti-bleaching solution (ThermoFisher P36935) and imaged by using LSM700 confocal microscope.

### Chromosome Spreading

Cells were blocked in metaphase using nocodazole at final concentration of 100 nM for 18 h. Cells were recovered and pelleted in cold PBS. Cells were resuspended in hypotonic buffer (75 mM KCl) during 15 min at 37°C, then transferred on ice and an equal volume of cold hypotonic solution containing 0.1% Tween20 was added in order to help spreading. Cells were immediately centrifuged in a cytofunnel chamber onto glass microscope slides during 5 min at 800 rpm. Slides were transferred to a coplin jar containing KCM solution (120 mM KCl, 20 mM Nacl, 10 mM Tris Hcl ph 7.5, 0.1% TritonX100) freshly made and incubated 7 min, then fixed 10 min in 2% paraformaldehyde. Slides were washed with KCM solution, staining with DAPI, and mounted with proLong gold anti-fade reagent and imaged by using LSM700 confocal microscope.

### Micrococcal Nuclease Digestion Assay

Control and patient lymphocytes were harvested at 1 × 10^6^ cells per time point. Nuclei were isolated by hypotonic lysis and micrococcal assay performed using the episcope nucleosome preparation kit (Takara, cat 5333) and following the instructions of the manufacturer. DNA was extracted using nucleospin PCR clean-up kit (Macherey-Nagel, ref 740609). DNA size and concentration were assessed by using the Fragment Analyzer Automated CE System (Advanced Analytical). We used the High Sensitivity NGS Fragment Analysis Kit DNF-474 and the PROSize 2.0 Software for accurate measurement.

### Statistical Analysis

Statistical analyses were conducted using Prism 6 software. Tests and *p*-values are indicated in figure legends.

## Data Availability Statement

The raw data supporting the conclusions of this manuscript will be made available by the authors, without undue reservation, to any qualified researcher.

## Ethics Statement

The studies involving human participants were reviewed and approved by IRB registration # 00001072, n°ID-RCB/EUDRACT:2015-A01170-49. Written informed consent to participate in this study was provided by the participants' legal guardian/next of kin. The animal study was reviewed and approved by Service de la performance, du financement et de la contractualisation avec les organismes de recherches. Comité d'éthique en expérimentation animale n 34.

## Author Contributions

M-TE-D and GS are the principal investigators. M-TE-D, CL, FG, FS, NL, CP, and GS performed the next-generation sequencing approaches, the functional *in vitro* studies and analyzed the results. JL, JB, J-SD, BN, AC-L'H, TM, and AF brought clinical and biological data. M-TE-D, AF, and GS wrote the paper. All authors revised and approved the final version of the manuscript.

### Conflict of Interest

The authors declare that the research was conducted in the absence of any commercial or financial relationships that could be construed as a potential conflict of interest.
