# Supplementary material for: Chronic Intestinal Pseudo-Obstruction and Lymphoproliferative Syndrome as a Novel Phenotype Associated With Tetratricopeptide Repeat Domain 7A Deficiency
Source: Front Immunol. 2019 Nov 7;10:2592. doi: 10.3389/fimmu.2019.02592 (PMC6853864; doi:10.3389/fimmu.2019.02592)
Supplement: Supplementary file 1 [file Data_Sheet_1.PDF]

Supplement to ***El-Daher M, et al.***

***Chronic intestinal pseudo-obstruction and lymphoproliferative syndrome as a novel phenotype associated with tetratricopeptide repeat domain 7A deficiency*.**

SUPPLEMENTARY FIGURES AND FIGURE LEGENDS

Supplementary Figure 1 H&E staining of the rectal biopsy performed at the age of 1 year

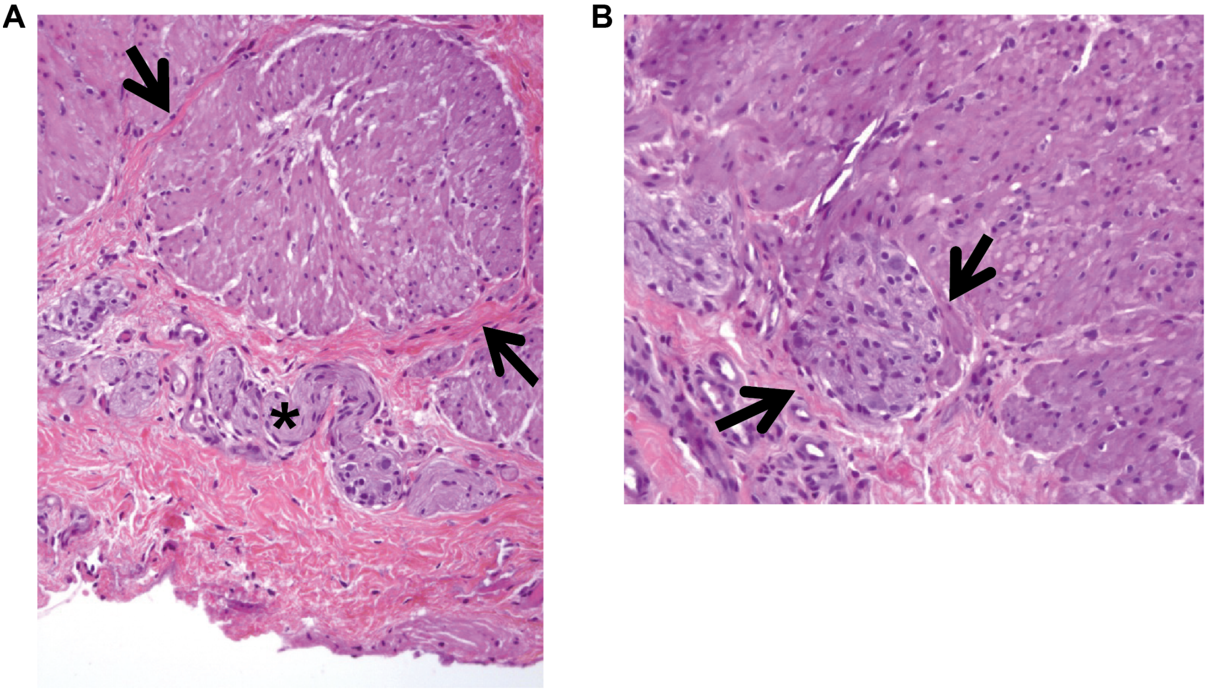

(A) Fibrosis with remodelling of the muscularis propria (arrow) with large myenteric plexus containing variable number of ganglionic cells were observed (*) without inflammatory ganglionitis

(B) Myenteric plexus were abnormally located within the deep part of the internal muscular layer

Supplementary Figure 2 Haplotype segregation in the P_R325Q patient family

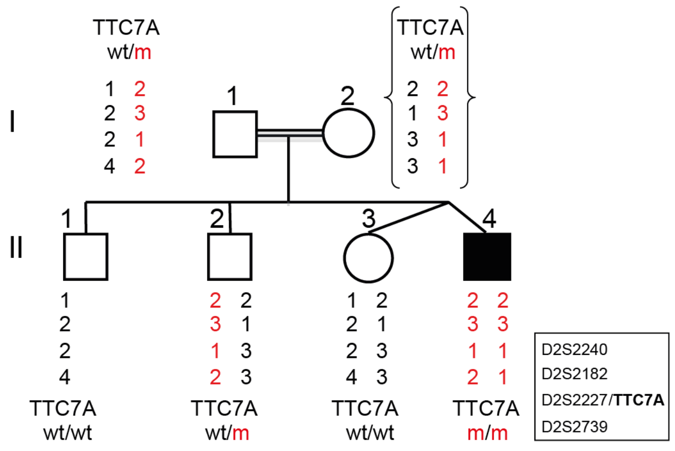

Family pedigree and genetic analysis showing the familial segregation of the polymorphic markers spanning the TTC7A wild-type (wt) gene and of the TTCA mutant identified (m). Data in brackets have been deduced for the unavailable mother

Supplementary Figure 3 Nuclear defects in T lymphoblast from R325Q_TTC7A patient

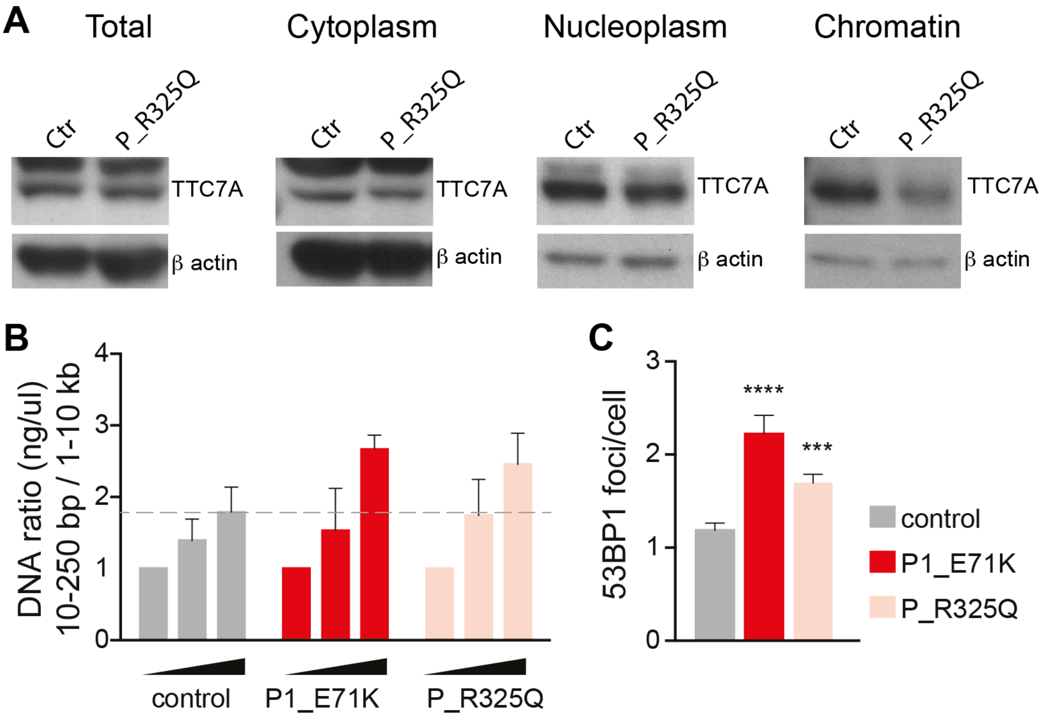

(A) TTC7A protein level is decreased in the chromatin fraction of primary T lymphoblast from R325Q_TTC7A (P_R325Q) patient as compared to healthy donor (Ctr)

(B) DNA accessibility is increased in T lymphoblast from P_R325Q and Ps-deficient patient (P1_E71K) as compared to control. The concentration of digested DNA was measured at different time points and the ratio between small and large size DNA fragments are shown in the graph.

(C) Increase DNA damage in patients' T lymphoblast as compared to control. Quantifications of the number of 53BP1 foci /cell are shown in the graph. Mean +/- SEM of 2 independent experiments. Total number of cells in control=508; P2_E71K n=315; P_R325Q n=452; Sidak's multiple comparisons test, ****p-value<0.0001

Supplementary Figure 4 Functional consequences of Ttc7a deficiency in *fsn* mouse

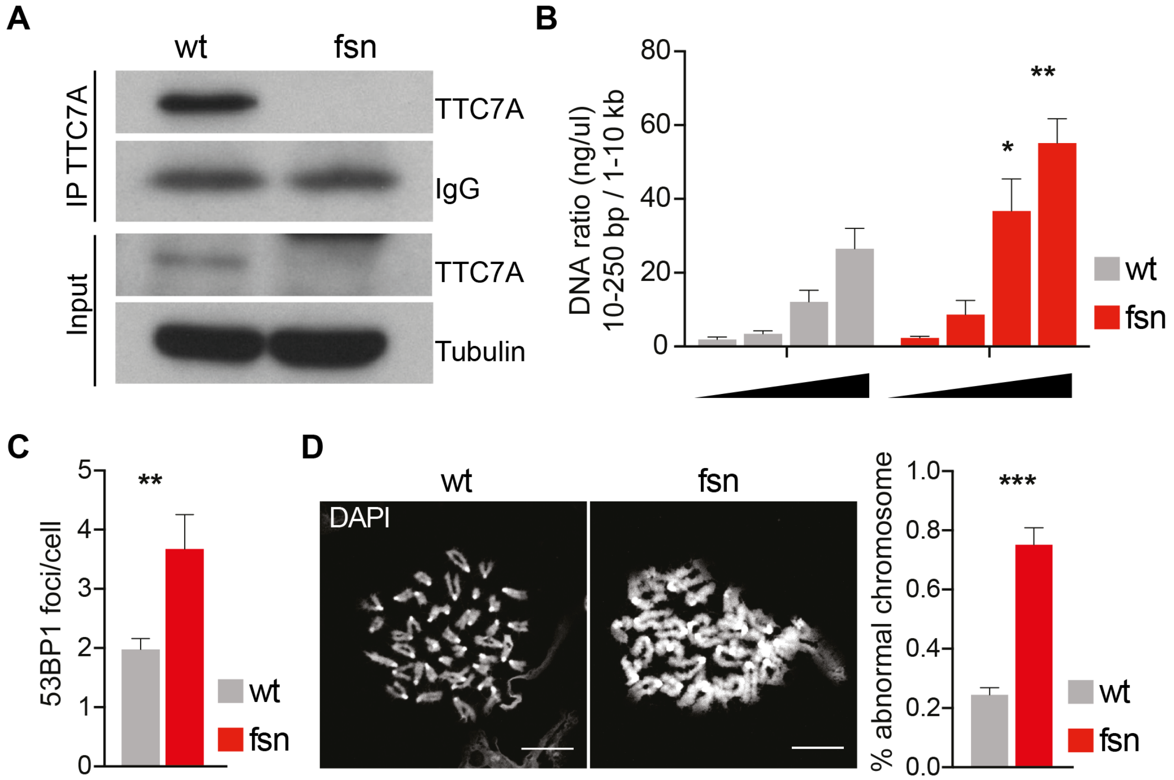

(A) Immunoprecipitation of Ttc7a protein from control (wt) and Ttc7a-deficient (*fsn*) bone marrow-derived dendritic cells (BMDC), by using TTC7A antibody. Immunoprecipitated and input proteins were resolved by western blot

(B) Increase kinetics of micrococcal nuclease digestion of chromatin in *fsn* T splenocytes as compared to wt. The concentration of digested DNA was measured at different time point and the ratio between small and large size DNA fragments are shown in the graph. Mean +/-SEM of 6 to 9 mice, multiple t-tests, *p-value=0.014087, **p-value=0.006237

(C) Accumulation of 53BP1 foci in *fsn* T splenocytes as compared to wt. Mean +/- SEM of 6 mice, paired t test, *p-value=0.0145

(D) Impaired chromatin condensation of *fsn* T splenocytes as compared to control. Left: DNA is stained with DAPI. Right: Quantification of chromosomes with abnormal structure. Mean +/-SEM of 4 mice, unpaired t tests, *** p-value=0.0002
